# Mediation Effects of IL-1β and IL-18 on the Association Between Vitamin D Levels and Mild Cognitive Impairment Among Chinese Older Adults: A Case–Control Study in Taiyuan, China

**DOI:** 10.3389/fnagi.2022.836311

**Published:** 2022-03-16

**Authors:** Le Cheng, Ruirui Dong, Chenmeng Song, Xuemin Li, Luping Zhang, Mengqian Shi, Chenhui Lv, Lili Wang, Jie Kou, Haoran Xie, Wenjuan Feng, Haifeng Zhao

**Affiliations:** ^1^Department of Nutrition and Food Hygiene, School of Public Health, Shanxi Medical University, Taiyuan, China; ^2^Center for Disease Control and Prevention in Shanxi Province, Taiyuan, China

**Keywords:** mild cognitive impairment, 25(OH)D3, inflammatory factors, IL-1β, IL-18, mediation effect, case-control study

## Abstract

**Objective:**

Mild cognitive impairment (MCI) is a common, chronic, and complex disease in the elderly, which is often influenced by a variety of factors that include nutrition and inflammation. This study was undertaken to evaluate the mediation effects of inflammation on the association between vitamin D levels and MCI.

**Methods:**

We explored the associations of inflammation and cognitive impairment related to 25(OH)D_3_ deficiency among 360 older people from the communities in China. Demographic characteristics, lifestyle, and health status were investigated by questionnaire, cognitive function was detected by MoCA, and plasma 25(OH)D_3_, interleukin-1β (IL-1β), and interleukin-18 (IL-18) were measured by ELISA. Spearman’s correlation analysis and logistic regression analysis were used to analyze the relationship among 25(OH)D_3_, IL-1β, and IL-18 in the MCI group and the control group and further to analyze the relationship between 25(OH)D_3_ and inflammatory factors in the MCI group. Finally, mediation analysis was performed to evaluate whether inflammation mediated the effect of 25(OH)D_3_ deficiency on cognitive impairment.

**Results:**

There were lower plasma 25(OH)D_3_ concentration and higher IL-1β and IL-18 levels in the MCI group compared with the controls. The levels of 25(OH)D_3_ were positively correlated with the MoCA scores and scores of different domains; the levels of IL-1β and IL-18 were negatively correlated with them (*p* < 0.05). In multivariate logistic analysis, there were significant associations among 25(OH)D_3_, IL-1β, IL-18, and MCI after adjusted. Further analysis revealed the significant association between the subjects with VD deficiency and the highest quartile of IL-18 in MCI (OR = 4.066), not with IL-1β after adjusting the confounding variables in MCI group. Ultimately, mediation analysis suggested that IL-1β and IL-18 could explain 25.4 and 17.5% of effect of the risk of cognitive impairment related to 25(OH)D_3_ deficiency.

**Conclusion:**

Our findings suggested that 25(OH)D_3_ deficiency could increase the risk of cognitive impairment by a mechanism partly involving inflammation. Therefore, vitamin D supplementation may improve or delay the decline in cognitive function caused by inflammation in the elderly.

## Introduction

Mild cognitive impairment (MCI) is characterized by a subtle decline in cognitive function and influenced by multiple factors that include pathophysiology, lifestyle, eating habits, and so on, and it is now also recognized as a risk factor for Alzheimer’s disease (AD) (Langa and Levine, 2014). In China, the latest report showed that the overall prevalence of dementia was 6.0% and of MCI was 15.5% in Chinese adults aged 60 years or older (Jia et al., 2020). This higher prevalence makes them an urgent public health problem in China, accompanied that the population has aged. But, the pathologic substrates of MCI and AD are equally complex and must take into account not only conventional the loss of neurons, plaque, and tangle pathology but also a wide range of cellular, biochemical, and molecular mechanisms, such as inflammation and so on (Mufson et al., 2012). Several population-based studies had also reported an association between peripheral inflammatory factors and the risk of MCI or dementia (Bossù et al., 2008; Scarabino et al., 2020). However, it is still unclear how much of the risk of cognitive impairment is caused by inflammation. Furthermore, there are also a lot of inflammation-related mechanisms, which also involve different inflammatory factors. Therefore, in this study, we selected interleukin-1β (IL-1β) and interleukin-18 (IL-18) as the inflammatory factors, which were not only related to inflammation, but also related to the loss of neurons.

Interleukin-1β and IL-18 can regulate or participate in neuronal damage through different mechanisms in the blood–brain barrier or in brain before or during AD occurred including in the brain and the periphery (Heppner et al., 2015; He et al., 2016; Heneka et al., 2018). Meanwhile, the high levels of IL-1β and IL-18 may be due to the activation of pyroptosis, a proinflammatory form of cell death. When pyroptosis has occurred, NOD-like receptors such as protein 1 (NLRP1), NLRP3, and other inflammasomes were activated by AD-related markers or cellular damage danger signals, which in turn activated caspase-1 and the pyroptosis execution protein gasdermin-D (GSDMD). These caused cell membrane damage and released large amounts of mature IL-1β and IL-18 (Heppner et al., 2015; He et al., 2016; Heneka et al., 2018).

Vitamin D (VD), as an essential micronutrient for the body, is mainly obtained through skin synthesis and dietary intake. Before exerting biological effects, VD must undergo hydroxylation in the liver and kidneys to be converted into 25-hydroxy-vitamin D [25(OH)D] and 1,25-dihydroxy-vitamin D [1,25(OH)_2_D] (Cui et al., 2017). As a neurosteroid hormone, VD also plays a certain neuroprotective effect in the brain. Some studies suggest that the lower 25(OH)D_3_ is strongly associated with cognitive decline and neurodegenerative disease (Brouwer-Brolsma and de Groot, 2015; Jones et al., 2015; Keeney and Butterfield, 2015). A meta-analysis found that serum 25(OH)D levels were significantly lower in subjects with MCI and dementia than in healthy controls (Annweiler et al., 2016). Some observational studies had been found that VD deficiency [25(OH)D < 25 ng/mL] and inflammation factors [interleukin-6 (IL-6), tumor necrosis factor-α (TNF-α), IL-1β, C-reactive protein (CRP)] had a significant correlation (Laird et al., 2014). Additionally, a study by Briones also found that in patients with AD, the serum levels of IL-1β and 25(OH)D_3_ showed a strong correlation (Briones and Darwish, 2012). Another study also explored the effect of VD supplementation on ameliorating cognitive function through the antiinflammatory mechanism *in vivo* (Medhat et al., 2020). Since deficiency in VD can be treated, VD may have an important public health inference in the prevention of age-related neurodegenerative diseases such as MCI and AD.

However, a challenge remains to fully understand the molecular mechanism of inflammation between VD and cognitive function. In other words, it was unclear what proportion of VD exerted neuroprotective effects through antiinflammatory mechanisms, or whether and how much the risk of increased cognitive impairment related to VD deficiency is explained by inflammation. Therefore, mediation analysis can be used to evaluate the mediating variable and further to reveal the internal mechanism and role of the causal association. So, we hypothesized that the 25(OH)D_3_ deficiency could increase the risk of cognitive impairment by a mechanism involving inflammation. To prove our hypothesis, we conducted a case–control study to detect the levels of VD and inflammatory factors in the elderly and analyze the correlation. We used mediation analysis to explore the potential role of inflammatory factors in the association between the levels of 25(OH)D_3_ and MCI.

## Materials and Methods

### Study Design and Participants

The data of the case–control study were obtained from a population-based epidemiological study on cognitive impairment among elderly population. The research subjects were local residents ≥65 years old who come from six major districts of Taiyuan, Shanxi, China. The interviews were conducted by face-to-face, and data collection and investigations were performed by trained staffs during the period from March 2016 to July 2017. Subjects were excluded from the study if they exhibited: cognitive dysfunction caused by other non-vascular factors such as ischemic cerebrovascular disease, systemic disease, taking drugs that affect cognitive function, degenerative disease, etc.; consciousness disturbances and patients with paranoia and mental illness; severe aphasia, hearing, visual impairment, severe movement, sensory impairment, etc.

All participants were informed of the objective of the study and their consent to participate in the study was obtained. The research protocol was approved by the Medical Ethics Committee of Shanxi Medical University, China.

### Sample Size

The population of this investigation was the elderly in the communities of Taiyuan city. The sample size was determined according to the sample size formula of the 1:1 ratio case–control study. According to the 2010–2013 China National Nutrition and Health Survey (CNNHS), the VD deficiency rate among the elderly was 39.15% (Chen et al., 2017). The odds ratio (OR) of the expected exposure to the research factor was estimated to be 2. The sample size was estimated for the two-sided test with error probabilities of α = 0.05 and 90% power (β = 0.10). Gender, age (±2 years), and education year (±2 years) as the matching factors and 180 aged people with MCI and 180 aged people with normal cognition served as the controls were included according to the calculation result.

### Data Collection

All subjects were interviewed with their caregivers present by trained interviewers. The questionnaire was designed to obtain the following information regarding the patients’ general characteristics: name, gender, age, height, weight, education level, whether to exercise, smoking, drinking, etc. The body mass index (BMI) based on the data of height and weight was calculated and divided into weight loss (<18.5), normal (18.5–23.9), overweight (24.0–27.9), and obesity (≥28.0) according to the Chinese adult BMI standard. The definition of education level was illiteracy, education period of 0–6 years, education period of 6 years or more. Smokers were defined as those who smoked at least 1 cigarette per day in the past 6 months or longer. Drinkers were who drank at least two times a week and drank continuously for more than one year. The definition of the exercise was in the past 6 months, at least five times a week, each time lasting at least 10 min or more of sports, exercise, or recreational activities.

### Assessment of Mild Cognitive Impairment

Participants underwent cognitive evaluation in a quiet room carried out by technicians with formal training. Montreal Cognitive Assessment (MoCA) was used to assess cognitive function, which includes the assessment of seven cognitive domains that include executive, naming, memory, attention, language, abstraction, and orientation (Ciesielska et al., 2016). The scoring standards are as follows: illiterate elderly with MoCA score ≤ 13 are classified as patients with MCI, ≥14 are classified as normal cognition; elderly people with education ≤ 6 years are classified as patients with MCI with MoCA score ≤ 19, and ≥20 are classified as normal cognition; elderly people with education years > 6 years with MoCA score ≤ 24 are classified as patients with MCI, ≥25 with normal cognition (Petersen, 2004).

### Inclusion Criteria for Case Group and Control Group

Case group: (1) the subjects were aged ≥ 65 years and were in good health; (2) the MoCA score belongs to patients with MCI; (3) Taiyuan residents with long-term residence in the urban area or suburb of Taiyuan city; (4) cognitive test can be completed.

Control group: (1) the age, gender, and education of the subjects were matched with case group, and they were healthy; (2) the MoCA score belonged to the elderly with normal cognition; (3) Taiyuan residents with long-term residence in the urban area or suburb of Taiyuan city; (4) cognitive test can be completed.

### Blood Sampling and Plasma Vitamin D and Inflammatory Factors Measurement

The blood samples were collected in the same season and drawn by venipuncture into 5-mL plain evacuated tubes and then centrifuged at 2,000 × g for 10 min from each participant after overnight fasting (8–12 h). All specimens were collected and analyzed within 1 h or stored at –80°C until use. Plasma vitamin D levels and levels of IL-1β and IL-18 were measured in duplicate using a commercially available enzyme-linked immunosorbent assay kit (Human 25-hydroxy vitamin D_3_, England; Human IL-1β and IL-18, Enzyme-linked Biotechnology, Shanghai, China) (Samochocki et al., 2013).

The international and Chinese recommended classification standards for the degree of VD deficiency are 25(OH)D_3_ < 10 ng/mL (<25 nmol/L) for severe deficiency, <20 ng/mL (<50 nmol/L) for deficiency, 21–29 ng/mL (52–72 nmol/L) is insufficient, and ≥30 ng/mL (≥75 nmol/L) is sufficient (de Oliveira et al., 2017).

### Statistical Analysis

**FIGURE 1:**
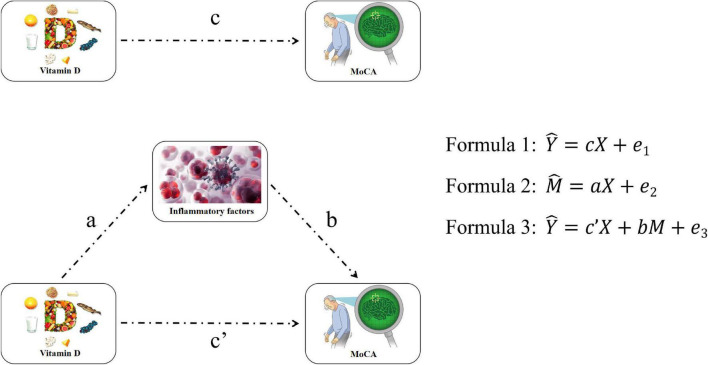
Flow of mediation effect analysis. *Y*, *X*, and *M* are MoCA scale score, VD, and inflammatory factors concentration, respectively. a, b, c, c′, e_1_, e_2_, and e_3_ are equation parameters.

Mean and standard deviations (SDs) or median (interquartile range) were used as descriptive statistics for continuous variables, and percentage was used for categorical variables. For continuous variables, the Student’s *t*-test or Mann–Whitney U test was used for between-group comparisons and chi-square test for categorical variables. Spearman’s correlation was conducted to analyze the relationship between VD, inflammatory factors, and cognitive impairment. Logistic regression was used to assess the association among VD, inflammatory factors, and risk of MCI. OR and corresponding 95% confidence intervals (CIs) were calculated. Model 1 was used to calculate the crude OR, and model 2 was adjusted age, gender, education, economic status, BMI, smoking status (yes/no), drinking status (yes/no), exercising status (yes/no), hypertension (yes/no), diabetes (yes/no), and hyperlipoidemia (yes/no). Furthermore, we conducted a mediating effect model to determine that the inflammatory factor changes could explain the cognitive impairment associated with VD status. The mediation effect analysis has used three linear equations to analyze the association among independent variables (VD), mediator variables (inflammatory factors), and dependent variables (MoCA). Variables such as age, gender, education, economic status, BMI, smoking, drinking, exercising, hypertension, diabetes, and hyperlipoidemia were included as confounders in the equation, as shown in Figure 1. The mediation proportion was used to evaluate the mediation effect in this study. All statistical analyses were performed using SPSS 22.0 and SAS 9.4. All reported *p*-values were two-sided and *p* < 0.05 were considered a statistically significant difference.

## Results

### Characteristics of Study Population

This study involved 180 aged people with MCI and 180 age-, sex- and education-matched controls. The mean age was approximately 73 years, with 50% being female sex. Table 1 shows the characteristics of the study participants. The results generally showed that there was no statistical difference between the two groups in terms of demographic characteristics, lifestyle, and health status (*p* > 0.05). Compared with the control group, the MCI group had lower scores in MoCA total scores and domains of executive, naming, memory, attention, language, abstraction, and orientation (*p* < 0.001). In this study, we found that 16 subjects (4.4%) were severe deficiency, 271 subjects (75.3%) were deficiency, 39 subjects (10.9%) were insufficient, and 34 subjects (9.4%) were sufficient about the level of plasma 25(OH)D_3_. There were fewer people in the MCI group who were sufficient, and more people who were insufficient, deficiency, and severe deficiency compared with the control group (*p* < 0.001).

**TABLE 1:** Characteristics of the study population.

Characteristics*		All subjects, *n* = 360	MCI, *n* = 180	Control, *n* = 180	*p-*Value*^#^*
Sex	Males	179 (49.7%)	90 (50.0%)	89 (49.4%)	0.916
	female	181 (50.3%)	90 (50.0%)	91 (50.6%)	
Age (year)		73.06 ± 5.53	73.33 ± 5.55	72.78 ± 5.52	0.342
Age	65–69	110 (30.6%)	53 (29.4%)	57 (31.6%)	0.316
	70–74	101 (28.0%)	46 (25.6%)	55 (30.6%)	
	75–79	108 (30.0%)	62 (34.4%)	46 (25.6%)	
	≥80	41 (11.4%)	19 (10.6%)	22 (12.2%)	
Educational level	illiteracy	43 (11.9%)	25 (13.9%)	18 (10.0%)	0.070
	<6 years	77 (21.4%)	30 (16.7%)	47 (26.1%)	
	≥6 years	240 (66.7%)	125 (69.4%)	115 (63.9%)	
BMI (kg/m^2^)		24.80 ± 4.86	24.34 ± 4.41	25.27 ± 5.25	0.068
BMI	<18.5	17 (4.7%)	12 (6.7%)	5 (2.8%)	0.131
	18.5–23.9	144 (40.0%)	77 (42.8%)	67 (37.2%)	
	24.0–27.9	141 (39.2%)	62 (34.4%)	79 (43.9%)	
	≥28.0	58 (16.1%)	29 (16.1%)	29 (16.1%)	
Physical activity	Yes	267 (74.2%)	134 (74.4%)	133 (73.9%)	0.904
	No	93 (25.8%)	46 (25.6%)	47 (26.1%)	
Smoking habit	Yes	102 (28.3%)	58 (32.2%)	44 (24.4%)	0.102
	No	258 (71.7%)	122 (67.8%)	136 (75.6%)	
Alcohol intake	Yes	68 (18.9%)	38 (21.1%)	30 (16.7%)	0.281
	No	292 (81.1%)	142 (78.9%)	150 (83.3%)	
Hypertension	Yes	164 (45.6%)	87 (48.3%)	77 (42.8%)	0.290
	No	196 (54.4%)	93 (51.7%)	103 (57.2%)	
Diabetes	Yes	54 (15.0%)	33 (18.3%)	21 (11.7%)	0.077
	No	306 (85.0%)	147 (81.7%)	159 (88.3%)	
Hyperlipemia	Yes	97 (26.9%)	51 (28.3%)	46 (25.6%)	0.553
	No	263 (73.1%)	129 (71.7%)	134 (74.4%)	
MoCA (scores)	Total score	21.21 ± 5.60	18.02 ± 5.05	25.65 ± 3.04	<0.001
	Executive	2.63 ± 1.66	1.91 ± 1.49	3.19 ± 1.02	<0.001
	Naming	2.67 ± 0.71	2.41 ± 0.90	2.89 ± 0.39	<0.001
	Memory	4.84 ± 1.56	4.29 ± 1.72	5.65 ± 0.70	<0.001
	Attention	2.03 ± 0.95	1.64 ± 1.02	2.54 ± 0.66	<0.001
	Language	1.31 ± 0.87	0.81 ± 0.87	1.61 ± 0.67	<0.001
	Abstraction	2.31 ± 1.37	1.26 ± 1.47	3.37 ± 1.38	<0.001
	Orientation	5.77 ± 0.61	5.53 ± 0.98	5.96 ± 0.22	<0.001
25(OH)D_3_	Severe deficiency	16 (4.4%)	15 (8.3%)	1 (0.6%)	<0.001
	Deficiency	271 (75.3%)	139 (77.2%)	132 (73.3%)	
	Insufficient	39 (10.9%)	24 (13.4%)	15 (8.3%)	
	Sufficient	34 (9.4%)	2 (1.1%)	32 (17.8%)	

*Results were shown as frequency percentages n (%) or mean ± SD. ^#^Independent sample t-test or Mann–Whitney U test was used for continuous variables. Pearson’s chi-squared was used for categorical variables.

### Comparisons of Plasma 25(OH)D3 and Inflammatory Factors Between Mild Cognitive Impairment and Control Groups

**FIGURE 2:**
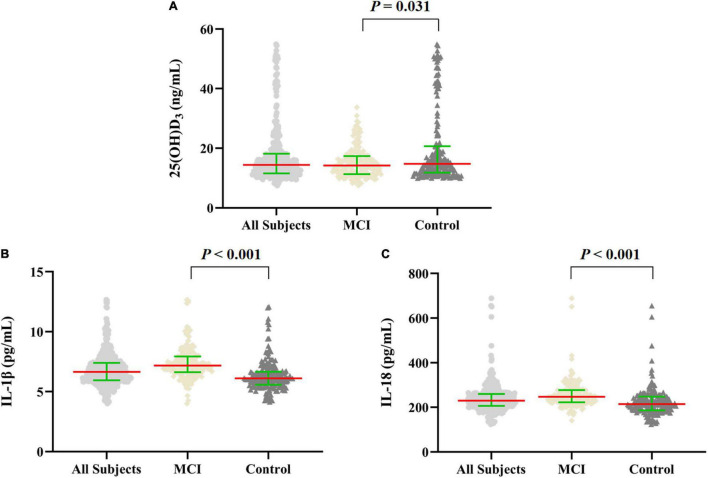
Scatter dot plot of the levels of plasma 25(OH)D_3_ and inflammatory factors in MCI and control groups. **(A–C)** The levels of plasma 25(OH)D_3_ and IL-1β, IL-18 in all subjects (*n* = 360), and in MCI and control groups (*n* = 180, respectively). The data were shown by the median with interquartile range, the red line represents the median, and the green line represents the interquartile range. Mann–Whitney *U* test was used for between-group comparisons.

In this case–control study, the median value of plasma 25(OH)D_3_ concentration of all subjects was 14.43 (11.58, 18.17) ng/mL, of which the median value of plasma 25(OH)D_3_ was 14.78 (11.81, 20.46) ng/mL in the control group, 14.16 (11.31, 17.38) ng/mL in the MCI group. Compared with the control group, the median value of plasma 25(OH)D_3_ concentration in the MCI group was lower (*p* = 0.031). Compared with the control group, the median values of plasma inflammatory factors IL-1β and IL-18 levels in the MCI group were significantly higher (*p* < 0.001; Figure 2).

### The Association Among Vitamin D, Inflammatory Factors, and Mild Cognitive Impairment

We used Spearman’s correlation and logistic regression to analyze the association among VD, inflammatory factors, and MCI (Tables 2, 3). Table 2 shows that the plasma 25(OH)D_3_ levels of the elderly were weak positively correlated with the total MoCA scores (*r* = 0.257) and were weak positively correlated with the language and abstraction (*r* = 0.121, 0.131, respectively). The plasma levels of IL-1β and IL-18 were negatively correlated with MoCA scores (*r* = −0.440, −0.434, respectively), and there was also a negative correlation in each domain (*p* < 0.001).

**TABLE 2:** Spearman’s correlation of the association among cognitive function, 25(OH)D_3,_ and inflammatory factors.

Variables	25(OH)D_3_ (ng/mL)	IL-1β (pg/mL)	IL-18 (pg/mL)
MoCA scores	0.257*	−0.440*	−0.434*
Executive	0.032	−0.303*	−0.341*
Naming	0.049	−0.246*	−0.277*
Memory	0.085	−0.239*	−0.206*
Attention	0.099	−0.274*	−0.288*
Language	0.121*	−0.282*	−0.296*
Abstraction	0.131*	−0.325*	−0.253*
Orientation	0.099	−0.239*	−0.230*

*Spearman’s correlation was used for the relationship between groups, p < 0.05.

**TABLE 3:** Logistic regression of the association among different cognitive status, 25(OH)D_3,_ and inflammatory factors.

Independent variables	Groups	Model 1			Model 2		
		OR_1_	95%CI_1_	*P*_1_	OR_2_	95%CI_2_	*P*_2_
25(OH)D_3_*	Q_1_	1.000	–	–	1.000	–	–
	Q_2_	9.289	3.180 ∼ 27.134	0.001	7.538	2.456 ∼ 23.136	0.000
	Q_3_	9.672	3.063 ∼ 30.544	0.000	9.111	2.729 ∼ 30.415	0.000
	Q_4_	15.725	4.880 ∼ 50.673	0.000	14.146	4.187 ∼ 47.795	0.000
	*P* for trend	0.004			0.016		
IL-1β^#^	Q_1_	1.000	–	–	1.000	–	–
	Q_2_	3.200	1.649 ∼ 6.210	0.001	4.221	2.031 ∼ 8.773	0.000
	Q_3_	6.909	3.531 ∼ 13.519	0.000	7.848	3.740 ∼ 16.465	0.000
	Q_4_	10.400	5.203 ∼ 20.786	0.000	12.619	5.889 ∼ 27.040	0.000
	*P* for trend	0.000			0.000		
IL-18^#^	Q_1_	1.000	–	–	1.000	–	–
	Q_2_	1.727	0.952 ∼ 3.133	0.072	1.532	0.804 ∼ 2.921	0.195
	Q_3_	1.652	0.911∼ 2.997	0.098	1.764	0.925 ∼ 3.361	0.085
	Q_4_	3.131	1.704 ∼ 5.752	0.000	3.373	1.736 ∼ 6.553	0.000
	*P* for trend	0.001			0.000		

*Model 1: Crude model.*

*Model 2: Adjusted for age, gender, education, economic status, BMI, smoking status (yes/no), drinking status (yes/no), exercising status (yes/no), hypertension (yes/no), diabetes (yes/no), and hyperlipoidemia (yes/no).*

*The groups of 25(OH)D_3_ status Q_1_ for sufficient; Q_2_ for insufficient; Q_3_ for deficiency; Q_4_ for severe deficiency.

*^#^The groups of IL-1β were divided by quartiles: Q_1_ for <25% (∼5.96 pg/mL); Q_2_ for 25–50% (5.98–6.65 pg/mL); Q_3_ for 50–75% (6.66–7.41 pg/mL); Q_4_ for >75% (∼7.44); the groups of IL-18 were divided by quartiles: Q_1_ for <25% (∼206.60 pg/mL); Q_2_ for 25–50% (206.71–229.88 pg/mL); Q_3_ for 50–75% (229.90–259.23 pg/mL); Q_4_ for >75% (∼259.49).*

Logistic regression was performed on the association among cognitive function, 25(OH)D_3_, and inflammatory factors. The crude (unadjusted) and adjusted ORs for the MCI according to the quartile concentrations of plasma 25(OH)D_3_, IL-1β, and IL-18 are shown in Table 3. In Model 1, compared with highest quartile of VD, lower quartiles of VD (Q_2_: OR_1_ = 9.289, 95% CI_1_: 3.180–27.134; Q_3_: OR_1_ = 9.672, 95% CI_1_: 3.063–30.544; Q_4_: OR_1_ = 15.725, 95% CI_1_: 4.880–50.673) were associated with MCI; compared with the lowest quartile, the higher quartiles of IL-1β (Q_2_: OR_1_ = 3.200, 95% CI_1_: 1.649–6.210; Q_3_: OR_1_ = 6.909, 95% CI_1_: 3.531–13.519; Q_4_: OR_1_ = 10.400, 95% CI_1_: 5.203–20.786) were associated with MCI, and the highest quartile of IL-18 (Q_4_: OR_1_ = 3.131, 95% CI_1_: 1.704–5.752) was associated with MCI. The significant association among VD, inflammatory factors, and MCI persisted after further adjusting for economic status, BMI, smoking, drinking, exercising, hypertension, diabetes, and hyperlipoidemia, with *p* for trend <0.05.

### The Relationship Between Vitamin D and Inflammatory Factors in Elderly Patients With Mild Cognitive Impairment

We further analyzed the association between the level of VD and inflammatory factors in the MCI group. Table 4 illustrates that the plasma 25(OH)D_3_ level was negatively correlated with the levels of IL-1β (*r* = −0.168, *p* = 0.025) and IL-18 (*r* = −0.257, *p* < 0.001). In multivariate logistic regression, the levels of IL-1β and IL-18 (quartiles) were used as dependent variables, and VD status (deficiency or not) was used as the independent variable to analyze, and it was found that either in the crude model or adjusted model, there was no correlation between VD and IL-1β (Table 5). In Model 1, there was no correlation between VD and IL-18 levels. After adjusting for age, gender, educational level, economic status, BMI, smoking, drinking, exercising, hypertension, diabetes, and hyperlipoidemia, the analysis found that compared with subjects with sufficient VD, the subjects with VD deficiency were associated with the highest quartile of IL-18 (Q_4_: OR_2_ = 4.066, 95% CI_2_: 1.654–9.995; Table 5).

**TABLE 4:** Spearman’s correlation of the association between 25(OH)D_3_ and inflammatory factors in mild cognitive impairment (MCI).

Inflammatory factors	25(OH)D_3_ (ng/mL)	
	*r*	*P*
IL-1β (pg/mL)	−0.168	0.025
IL-18 (pg/mL)	−0.257	<0.001

**TABLE 5:** Logistic regression of the association between vitamin D (VD) deficiency and inflammatory factors in MCI group.

Dependent variables	Groups	Model 1			Model 2		
		OR_1_	95%CI_1_	*P*_1_	OR_2_	95%CI_2_	*P*_2_
IL-1β*	Q_1_	1.000	–	–	1.000	–	–
	Q_2_	0.773	0.285–2.096	0.163	0.508	0.159–1.629	0.255
	Q_3_	3.132	0.713–12.073	0.069	2.424	0.511–11.502	0.265
	Q_4_	3.500	0.880–13.918	0.075	2.790	0.637–12.216	0.173
IL-18*	Q_1_	1.000	–	–	1.000	–	–
	Q_2_	1.444	0.468–3.732	0.599	0.995	0.509–1.943	0.987
	Q_3_	2.235	0.612–5.637	0.274	1.681	0.831–3.398	0.148
	Q_4_	5.090	0.777–13.403	0.094	4.066	1.654–9.995	0.002

*Model 1: Crude model.*

*Model 2: Adjusted for age, gender, education, economic status, BMI, smoking status (yes/no), drinking status (yes/no), exercising status (yes/no), hypertension (yes/no), diabetes (yes/no), and hyperlipoidemia (yes/no).*

*The groups of IL-1β were divided by quartiles: Q_1_ for <25% (∼5.96 pg/mL); Q_2_ for 25–50% (5.98–6.65 pg/mL); Q_3_ for 50–75% (6.66–7.41 pg/mL); Q_4_ for >75% (∼7.44); the groups of IL-18 were divided by quartiles: Q_1_ for <25% (∼206.60 pg/mL); Q_2_ for 25–50% (206.71–229.88 pg/mL); Q_3_ for 50–75% (229.90–259.23 pg/mL); Q_4_ for >75% (259.49∼).

### Mediate Effects of Inflammatory Factors on the Association Between 25(OH)D3 and Cognition

We performed mediation analysis of inflammatory factors in the association between 25(OH)D_3_ and cognitive status. We observed significant mediation effects of IL-1β and IL-18 in the association between 25(OH)D_3_ and cognition in Table 6 (*p* = 0.013, 0.004, respectively). The mediation analysis showed a mediation proportion of 25.4% (95% CI: 9.2–53.2%) in IL-1β and a mediation proportion of 17.5% (95% CI: 7.2–36.7%) in IL-18. These results suggested that IL-1β and IL-18 may be the potential mediators of 25(OH)D3 deficiency effect on the risk of cognitive impairment.

**TABLE 6:** Mediate effects of inflammatory factors in the association between 25(OH)D_3_ and cognitive status.

Variables	IL-1β		IL-18	
	Proportion mediated*	*p-*Value	Proportion mediated*	*p*-Value
MoCA scores	25.4% (9.2–53.2%)	0.013	17.5% (7.2–36.7%)	0.004
Executive	–	–	–	–
Naming	–	–	34.1% (7.4–76.9%)	0.012
Memory	16.6% (5.4–40.7%)	0.016	–	–
Attention	34.6% (8.7–74.6%)	0.012	22.2% (6.2–55.4%)	0.007
Language	33.4% (8.1–74.0%)	0.010	22.0% (4.8–61.4%)	0.029
Abstraction	21.1% (5.6–54.5%)	0.026	14.7% (3.9–42.1%)	0.030
Orientation	–	–	–	–

*Covariates in the SAS macro include age, gender, education, economic status, BMI, smoking status (yes/no), drinking status (yes/no), exercising status (yes/no), hypertension (yes/no), diabetes (yes/no), and hyperlipoidemia (yes/no).

After further analysis of the various domains of cognition, the results found that IL-1β had significant mediation effects in the association between 25(OH)D_3_ and memory, attention, language, and abstraction (*p* < 0.05), and IL-18 had significant mediation effects in the association between 25(OH)D_3_ and naming, attention, language, and abstraction (*p* < 0.05).

## Discussion

In this study, we observed the lower plasma 25(OH)D_3_ concentration and higher IL-1β and IL-18 levels in the MCI group, and there were significant associations among 25(OH)D_3_, inflammatory factors, and MCI. Significantly, after the mediation analysis, we also found that the 25(OH)D_3_ deficiency could increase the risk of cognitive impairment by a mechanism partly involving inflammation, which could explain 25.4 (IL-1β) and 17.5% (IL-18) of effect of the risk of cognitive impairment related to 25(OH)D_3_ deficiency, and be also applicable to different domains of cognition.

Plasma 25(OH)D_3_ is determined by endogenous vitamin D synthesis and/or dietary intake, conversion into 25(OH)D_3_, and, finally, distribution and usage (metabolism and excretion). The concentration of 25(OH)D_3_ in plasma is largely unregulated, and it has a relatively long half-life of 2–3 weeks (Cui et al., 2017). Therefore, in this study, we assessed the VD nutritional status by detecting plasma 25(OH)D_3_, which is the most commonly used marker of vitamin D status. In this study, we found that the median value of plasma 25(OH)D_3_ concentration of all subjects was 14.43 ng/mL, and 16 subjects (4.4%) were severe deficiency, 271 subjects (75.3%) were deficiency, 39 subjects (10.9%) were insufficient, and 34 subjects (9.4%) were sufficient about the level of plasma 25(OH)D_3_. The constituent ratio of 25(OH)D_3_ deficiency in this study was higher than that of 25(OH)D_3_ deficiency (39.2%) in CNNHS (Chen et al., 2017). We further explored the differences in age composition, seasons, and residences of the two surveys. It turned out that CNNHS survey results showed that 25(OH)D_3_ deficiency was positively correlated with the spring season, low ambient UVB levels, and living in large cities (Chen et al., 2017). The Taiyuan city in Shanxi Province that we surveyed belongs to low ambient UVB levels and large cities, and the time when we did our survey and the collection time of blood samples were both in spring. In addition, the age among our subjects was older than CNNHS, which may be the reason why the constituent ratio of 25(OH)D_3_ deficiency in our results was higher.

As a neurosteroid hormone, VD also has a certain neuroprotective effect on the brain. When VD deficiency occurred, it may be brought about cognitive dysfunction, cognitive decline, or neurodegenerative diseases, and so on (Brouwer-Brolsma and de Groot, 2015; Jones et al., 2015; Keeney and Butterfield, 2015). Several previous observational studies had reported an association between low levels of serum vitamin D and MCI or dementia in the elderly (Goodwill and Szoeke, 2017; Overman et al., 2017). In our research, focusing on the MCI population, we also found that plasma 25(OH)D_3_ level was significantly decreased in MCI compared with the control group. Furthermore, plasma 25(OH)D_3_ concentration was positively correlated with MoCA scores.

The underlying mechanisms of the association between 25(OH)D_3_ deficiency and cognitive impairment remain an open question, and it may be related to the role of VD in the brain. 25(OH)D_3_ and 1,25(OH)_2_D_3_ could regulate the survival, development, and function of neural cells (Annweiler et al., 2013). VD also could reduce amyloid-induced cytotoxicity and apoptosis in primary cortical neurons (Mizwicki et al., 2012). Additionally, vitamin D supplementation ameliorates age-related decline in learning and memory in aged rats and this may be one of the measures to prevent or delay cognitive impairment (Briones and Darwish, 2012). The preventive effect of VD may be exerted through an antiinflammatory mechanism (Laird et al., 2014). The level of IL-1β was increased in AD during 25(OH)D_3_ was deficient and the effect of VD supplementation on ameliorating cognitive function through the antiinflammatory mechanism *in vivo* (Medhat et al., 2020).

Inflammation has been confirmed to be involved in the pathogenesis and progression of AD. Indeed, inflammatory processes play at least some roles in the pathology of AD and MCI. Particularly, intriguing are peripheral inflammatory cytokines, studies had found that the level of peripheral inflammatory cytokines in AD has reached its peak in the early stage of the disease, which may precede the clinical symptoms of AD (Motta et al., 2007). Moreover, the researchers found a third of patients with MCI remained as they were, a third reversed diagnosis to cognitively normal, thereby providing a large time “critical window” to the prevention (Manly et al., 2008). In this study, we may pay more attention to how much of the risk of cognitive impairment is caused by inflammation in this “critical window” to prevention. We selected IL-1β and IL-18 in the peripheral blood of patients with MCI as the inflammatory factors, which were not only related to the inflammation, but also related to the loss of neurons which as the typical pathological characteristics of MCI and AD. The high levels of inflammatory factors in the periphery and brain would further invade the brain and neurons, which make the condition worse. Our research found that compared with the control group, the levels of plasma IL-1β and IL-18 in MCI were significantly higher, and IL-1β and IL-18 were negatively correlated with MoCA scores and scores of different domains. After further regression analysis, changes in two inflammatory factors were found, so it was speculated that systemic inflammation might be a risk factor for MCI. These higher levels of IL-1β and IL-18 may further remind us that, in the “critical window period,” the detection of the levels of peripheral inflammatory factors in patients with MCI may play a certain role in the prognosis of disease.

As mentioned above, the previous studies reported that in patients with AD, the serum levels of IL-1β and 25(OH)D_3_ showed a strong negative correlation (Briones and Darwish, 2012). In this study, we found that the plasma 25(OH)D_3_ level was negatively correlated with the levels of IL-1β and IL-18, and VD deficiency may be a risk factor for high levels of inflammatory factors in MCI. Interestingly, however, we only found the role for IL-18, not both. This may be related to the production of IL-18. IL-18 can be produced by chondrocytes, osteoblasts, and macrophages in joints and also present in keratinocytes and nearly all epithelial cells (Dinarello et al., 2013). Additionally, these are also the main ways that VD plays the physiological functions *in vivo*. Furthermore, we further analyzed whether and how much the risk of increased cognitive impairment related to VD deficiency was explained by inflammation. The mediation analysis showed that IL-1β and IL-18 could explain 25.4 and 17.5% of effect of the risk of cognitive impairment related to 25(OH)D_3_ deficiency. In the analysis of different cognitive domains, it was found that IL-1β and IL-18 may have some relationships in attention, language, and abstraction. IL-1β was significantly related to memory, while IL-18 was significantly related to naming. But both have nothing to do with executive and orientation. Although our study found these interesting results, an interleukin is responsible for specific cognitive alteration that is seemly unrealistic. Therefore, we still need to conduct a lot of research to further explore or confirm them.

The following limitations of this study should be considered. Whether low vitamin D concentrations play a causal role in the pathogenesis of a cognitive disease or are the consequences of an inadequate intake secondary to the illness remains an open issue. Thus, further longitudinal studies and randomized controlled trials are needed to examine the temporal sequence of this association. Although we matched the confounding factors, the results of this case–control study may still be misinterpreted because of the influence of random and systematic recall errors, and selection bias. However, our research answers the mediation effects of inflammation on the association between VD and MCI partially and provides certain population data support for the follow-up study of the antiinflammatory mechanism of VD. At the same time, it also plays a certain value in the prevention and delay of cognitive impairment.

## Conclusion

In summary, this study not only demonstrated that the elderly individuals with MCI presented decreased plasma vitamin D levels and increased IL-1β and IL-18 concentrations, and there were significant associations among 25(OH)D_3_, inflammatory factors, and MCI. Furthermore, it was revealed that the 25(OH)D_3_ deficiency could increase the risk of cognitive impairment by a mechanism partly involving inflammation. Although further prospective larger studies and rigorous animal or cell experiments should be conducted to examine the association between vitamin D and the risk of cognitive decline and to clarify and verify whether this association may be caused by systemic inflammation. This study still provides some nutritional intervention strategies for preventing cognitive decline in the elderly and hopes to lay a certain research foundation for the realization of “healthy aging.”

## Data Availability Statement

The original contributions presented in the study are included in the article/supplementary material, further inquiries can be directed to the corresponding author/s.

## Ethics Statement

The studies involving human participants were reviewed and approved by the Medical Ethics Committee of Shanxi Medical University, China (protocol code 2014030 and date of approval 7th March, 2014). The patients/participants provided their written informed consent to participate in this study.

## Author Contributions

LC contributed to the execution of the experiment. RD, CS, XL, and LZ contributed to the acquisition, analysis, and interpretation of data. MS contributed to the creation of new software used in the work. CL, LW, JK, HX, and WF contributed to the evaluation, analysis, and wrote the data. HZ contributed to the guidance and substantive revision of the drafting work. LC and HZ take full responsibility for the contents of the manuscript, agree to be personally accountable for the author’s own contributions, for ensuring that questions related to the accuracy or integrity of any part of the work, even ones in which the author was not personally involved, are appropriately investigated, resolved, and documented in the literature. All authors performed revisions of the manuscript, contributed to the article, and approved the submitted version.

## Conflict of Interest

The authors declare that the research was conducted in the absence of any commercial or financial relationships that could be construed as a potential conflict of interest.

## Publisher’s Note

All claims expressed in this article are solely those of the authors and do not necessarily represent those of their affiliated organizations, or those of the publisher, the editors and the reviewers. Any product that may be evaluated in this article, or claim that may be made by its manufacturer, is not guaranteed or endorsed by the publisher.
